# UK5099 Inhibits the NLRP3 Inflammasome Independently of its Long‐Established Target Mitochondrial Pyruvate Carrier

**DOI:** 10.1002/advs.202307224

**Published:** 2024-07-01

**Authors:** Linyu Ran, Miao Chen, Jihui Ye, Song Zhang, Zhibing Luo, Tengfei Bai, Chenchen Qian, Quan Zhou, Mengtian Shan, Yong Chu, Joerg Herrmann, Qiang Li, Feilong Wang

**Affiliations:** ^1^ Department of Pulmonary and Critical Care Medicine Shanghai East Hospital Tongji University Shanghai 200120 China; ^2^ Medical College Tongji University Shanghai 200092 China; ^3^ Department of Emergency The First Affiliated Hospital of Hainan Medical University Haikou Hainan 570102 China; ^4^ Department of Cardiovascular Medicine Mayo Clinic Rochester MN 55902 USA; ^5^ Center for Regenerative Medicine Mayo Clinic Rochester MN 55902 USA; ^6^ Department of Medicinal Chemistry School of Pharmacy Fudan University 826 Zhangheng Rd Shanghai 201203 China; ^7^ Division of Hospital Internal Medicine Mayo Clinic Phoenix AZ 85054 USA

**Keywords:** macrophage, metabolism, mitochondrial pyruvate carrier, NLRP3, UK5099

## Abstract

Targeting NLRP3 inflammasome has been recognized as a promising therapeutic strategy for the treatment of numerous common diseases. UK5099, a long‐established inhibitor of mitochondrial pyruvate carrier (MPC), is previously found to inhibit macrophage inflammatory responses independent of MPC expression. However, the mechanisms by which UK5099 inhibit inflammatory responses remain unclear. Here, it is shown that UK5099 is a potent inhibitor of the NLRP3 inflammasome in both mouse and human primary macrophages. UK5099 selectively suppresses the activation of the NLRP3 but not the NLRC4 or AIM2 inflammasomes. Of note, UK5099 retains activities on NLRP3 in macrophages devoid of MPC expression, indicating this inhibitory effect is MPC‐independent. Mechanistically, UK5099 abrogates mitochondria‐NLRP3 interaction and in turn inhibits the assembly of the NLRP3 inflammasome. Further, a single dose of UK5099 persistently reduces IL‐1β production in an endotoxemia mouse model. Importantly, structure modification reveals that the inhibitory activities of UK5099 on NLRP3 are unrelated to the existence of the activated double bond within the UK5099 molecule. Thus, this study uncovers a previously unknown molecular target for UK5099, which not only offers a new candidate for the treatment of NLRP3‐driven diseases but also confounds its use as an MPC inhibitor in immunometabolism studies.

## Graphical abstract

UK5099, the gold standard mitochondrial pyruvate carrier (MPC) inhibitor, is found to selectively suppress the activation of the NLRP3 inflammasome in both mouse and human primary macrophages as well as in an endotoxemia mouse model. These effects are independent of its activity on MPC and is unrelated to the existence of the activated double bond within the molecule.

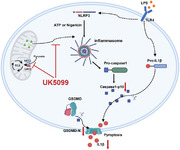

## 1 Introduction

The NLRP3 inflammasome is a cytosolic protein complex that contains sensor molecule NOD‐like receptor pyrin domain‐containing 3 (NLRP3), the adaptor protein apoptosis‐associated speck‐like protein containing a CARD (ASC) and the effector caspase‐1.^[^ ^1^, ^2^ ^]^ As one of the best characterized inflammasomes, the NLRP3 inflammasome has been recognized as a key factor in regulating host defense in response to a broad range of stimuli, including pathogen‐derived and endogenous agents.^[^ ^2^ ^]^ Stimulation of the NLRP3 inflammasome leads to ASC assembly and the autocatalytic activation of caspase‐1, which in turn cleaves the proinflammatory cytokines IL‐1β and IL‐18 and induces gasdermin‐D‐dependent cell death, known as pyroptosis.^[^ ^1^, ^2^ ^]^ Over the past two decades, NLRP3 inflammasome‐mediated immune responses have been linked to a wide variety of diseases, including type 2 diabetes, atherosclerosis, Alzheimer's disease, gout and most recently coronavirus disease (COVID‐19) with pathophysiological and therapeutic implications.^[^ ^3^, ^4^, ^5^, ^6^, ^7^ ^]^ Indeed, inhibition of the NLRP3 inflammasome has been considered as a promising therapeutic approach for the treatment of numerous common diseases.^[^ ^8^ ^]^ However, to date, there is no approved NLRP3 inflammasome inhibitor for human use, despite some chemical compounds having been developed that are currently undergoing preclinical or clinical trials.^[^ ^9^, ^10^ ^]^ Therefore, searching for new NLRP3 inhibitors is still a pressing need.

The small molecule α‐cyano‐β‐(1‐phenylindol‐3‐yl)‐acrylate, also known as UK5099, inhibits mitochondrial pyruvate carrier (MPC), which mediates the transport of pyruvate into the mitochondria.^[^ ^11^ ^]^ UK5099 was originally synthesized in 1970s as the analog of α‐cyano‐4‐hydroxycinnamate and was shown to effectively reduce the entry of pyruvate into mitochondria isolated from rat liver and hearts.^[^ ^12^, ^13^ ^]^ The primary target of UK5099 was subsequently recognized as MPC after two independent groups identified this hetero‐oligomeric complex of proteins in 2012.^[^ ^14^, ^15^ ^]^ As the most potent MPC inhibitor described to date, UK5099 has been considered a gold standard for MPC inhibition and has been widely used in various metabolism‐related studies over many years, especially in the field of immunometabolism.^[^ ^11^, ^16^, ^17^, ^18^, ^19^, ^20^, ^21^, ^22^ ^]^ Indeed, many publications have validated the importance of MPC‐mediated metabolism in the biological function of various types of immune cells based on the use of UK5099, with very few of them confirmed by genetic approaches.^[^ ^16^, ^17^, ^18^, ^19^, ^20^, ^21^ ^]^ Little attention has been paid to possible other targets of this chemical compound, and in a previous study, we found that the inhibitory effect of UK5099 on macrophage inflammatory responses does not match its activity on MPC.^[^ ^23^ ^]^ Further, UK5099 equally reduced the production of proinflammatory cytokines in wild‐type (WT) macrophages and macrophages that lack MPC expression, indicating the existence of unknown off‐target(s) of UK5099.^[^ ^23^ ^]^ However, the molecular target(s) of UK5099 other than MPC remains unknown. A better understanding of on‐ and off‐targets of UK5099 is not only important to properly interpret the findings of previous studies but also required to avoid any confounding findings involving its use in future studies.

Here, we found that UK5099 inhibits the activation of the NLRP3 inflammasome in both mouse and human primary macrophages independently of its established target MPC. Moreover, UK5099 effectively inhibits the production of IL‐1β in an endotoxemia mouse model. Therefore, in addition to MPC inhibition, UK5099 is a specific and potent inhibitor of NLRP3 inflammasome and could serve as a potential drug candidate for the treatment of NLRP3‐driven inflammatory diseases.

## 2 Results

### 2.1 UK5099 Inhibits NLRP3 Inflammasome‐Driven IL‐1β Release and Pyroptotic Cell Death

**Figure 1:**
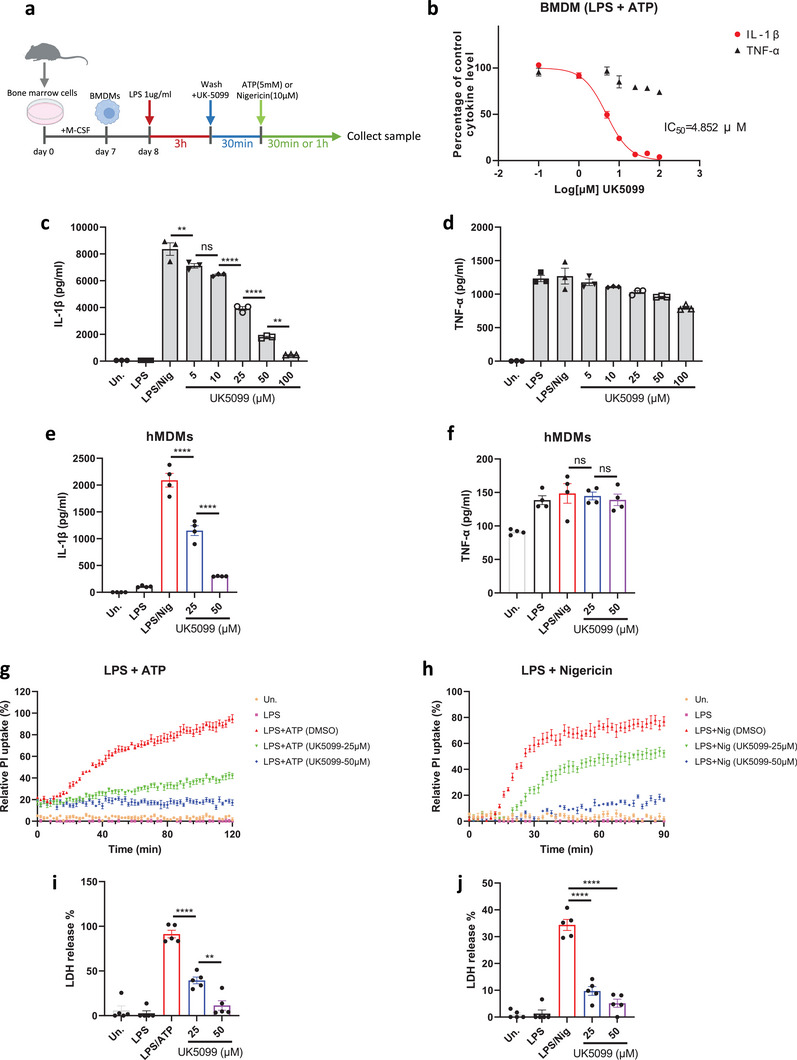
UK5099 inhibits NLRP3‐driven IL‐1β release and pyroptotic cell death. a). A schematic diagram illustrating the design of the NLRP3 inflammasome assay with UK5099 in mouse derived BMDMs (also applicable to hMDMs). b). Production of IL‐1β and TNF‐α from BMDMs stimulated with LPS and ATP and treated with UK5099 (0–100 µM). Data are representative of four independent experiments (*n* = 3, mean ± SEM). c,d). Production of IL‐1β (c) and TNF‐α (d) from BMDMs stimulated with LPS and nigericin and treated with different concentrations of UK5099. Data are representative of three independent experiments (*n* = 3, mean ± SEM). e,f). Production of IL‐1β (e) and TNF‐α (f) from hMDMs stimulated with LPS and nigericin and treated with different concentrations of UK5099 (*n* = 4, mean ± SEM). g,h). The uptake of PI as measured kinetically in BMDMs stimulated with LPS and ATP (g) or nigericin (h) and treated with different concentrations of UK5099. Data are representative of three independent experiments (*n* = 6, mean ± SEM). i,j). Production of LDH from BMDMs stimulated with LPS and ATP (i) or nigericin (j) and treated with different concentrations of UK5099. Data are representative of three independent experiments (*n* = 5, mean ± SEM). One way ANOVA followed by Tukey's test was used to compare multiple groups (c,e,f,i and j). Differences were considered statistically significant at ^**^ *P* ≤ 0.01 and ^****^ *P* < 0.0001; NS, no significant difference.

As UK5099 reduces macrophage inflammatory responses independent of its well‐known target MPC,^[^ ^23^ ^]^ we tested if it affects the activation of NLRP3 inflammasome, which is a vital player in innate immunity and inflammation. To this end, we employed well‐established LPS plus ATP stimulation protocols to activate the NLRP3 inflammasome.^[^ ^9^, ^10^, ^24^, ^25^ ^]^ Mouse bone marrow‐derived macrophages (BMDMs) were primed with LPS for 3 hours, followed by treatment with different concentrations of UK5099 prior to activation of NLRP3 with ATP (**Figure** **1**a). UK5099 reduced the release of IL‐1β in BMDMs in a dose‐dependent manner, with a half‐maximal inhibitory concentration (IC_50_) of approximately 4.85 µM (Figure 1b). Tumor necrosis factor‐α (TNF‐α) production was not reduced by UK5099 in the same setting (Figure 1b), indicating that the inhibitory effect of UK5099 on IL‐1β secretion is specific. Similarly, treatment of LPS‐primed BMDMs with UK5099 effectively suppressed IL‐1β release, but not TNF‐α production, after stimulation with nigericin, which is another well‐known activator of the NLRP3 inflammasome (Figure 1c and 1d). Importantly, UK5099 also inhibited IL‐1β release, but not TNF‐α production, in human monocyte‐derived macrophages (hMDMs), suggesting that UK5099 could block NLRP3 inflammasome activation across different species (Figure 1e and 1f).

In addition to IL‐1β maturation, NLRP3 inflammasome activation also leads to the cleavage of gasdermin D, which, in turn, triggers pore formation in the cell membrane to actuate a form of programmed cell death known as pyroptosis.^[^ ^1^, ^2^ ^]^ We then tested whether UK5099 could inhibit NLRP3‐driven pyroptosis and found that it dose‐dependently reduced the formation of pyroptotic pores, as measured by propidium iodide (PI) uptake (Figure 1g and 1h). In addition, UK5099 also reduced pyroptotic cell death, as measured by lactate dehydrogenase (LDH) release (Figure 1i and 1j). Taken together, UK5099 effectively inhibits NLRP3‐driven IL‐1β production and pyroptotic cell death.

### 2.2 UK5099 Blocks ASC Oligomerization and the Cleavage of Caspase‐1, IL‐1β and Gasdermin D

**Figure 2:**
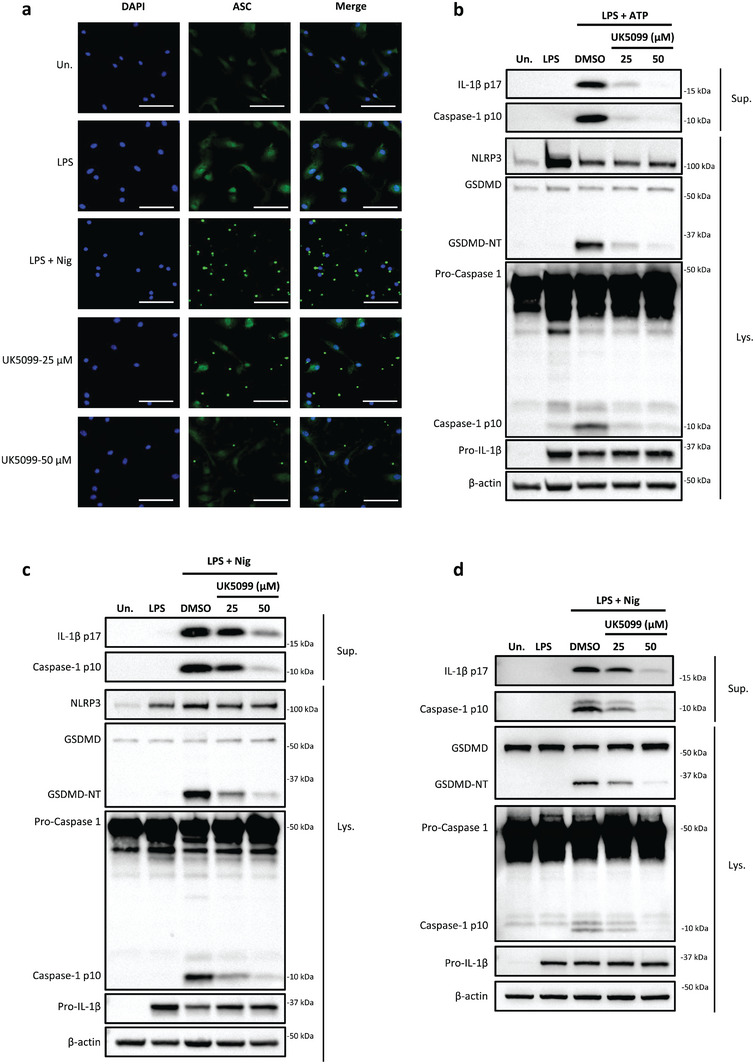
UK5099 blocks NLRP3‐dependent ASC oligomerization and the cleavage of pro‐caspase‐1 and gasdermin D. a). Live cell imaging of ASC‐citrine BMDMs stimulated with LPS and nigericin and treated with different concentrations of UK5099. The images are representative of three independent experiments. (Scale bar, 50 mm). b) Western blots of cell lysates (Lys.) and supernatants (Sup.) from BMDMs stimulated with LPS and ATP and treated with different concentrations of UK5099. Data are representative of three independent experiments. c) Western blots of cell lysates (Lys.) and supernatants (Sup.) from BMDMs stimulated with LPS and nigericin and treated with different concentrations of UK5099. Data are representative of three independent experiments. d) Western blots of cell lysates (Lys.) and supernatants (Sup.) from hMDMs stimulated with LPS and nigericin and treated with different concentrations of UK5099. Data are representative of three independent experiments.

Upon activation, NLRP3 recruits ASC to form large multimeric complexes, known as ASC specks, to active caspase‐1 and to subsequently cleave IL‐1β and gasdermin D.^[^ ^1^, ^2^ ^]^ To test the impact of UK5099 on ASC speck formation, we employed BMDMs expressing ASC fluorescent protein (ASC‐citrine), which permits the visualization of ASC oligomerization.^[^ ^26^ ^]^ We found that ASC fluorescence was uniformly distributed in resting macrophages and the formation of compact specks were apparent upon stimulation with LPS plus nigericin (**Figure** **2**a). Strikingly, UK5099 dose‐dependently decreased the formation of ASC specks (Figure 2a), indicating the NLRP3‐recruited ASC aggregation was blocked.

We next tested the effect of UK5099 on the downstream signaling of ASC speck assembly. While UK5099 had no impact on the expression of pro‐IL‐1β, it substantially reduced the cleavage of pro‐IL‐1β and pro‐caspase‐1 to their mature p17 and p10 forms in BMDMs upon LPS plus ATP or nigericin stimulation (Figure 2b,c). Moreover, UK5099 reduced cleavage of gasdermin D into its active N‐terminal fragment, which is the executor of pyroptosis (Figure 2b,c). Importantly, these observations were also confirmed in hMDMs (Figure 2d).

### 2.3 UK5099 Inhibits NLRP3 Inflammasome Activation Independently of MPC

The well‐established target of UK5099 is MPC.^[^ ^11^, ^16^ ^]^ Although we previously demonstrated that UK5099 inhibits a macrophage inflammatory response independently of MPC‐mediated metabolism,^[^ ^23^ ^]^ it is unknown if its effect on NLRP3 assembly and activity is related to its inhibition of MPC. To answer this question, we first tested if other MPC inhibitors could block the activation of the NLRP3 inflammasome. Mitoglitazone (MSDC‐0160) is a second‐generation thiazolidinedione, which was identified as a potent MPC inhibitor and has completed promising Phase 2 clinical trials for type 2 diabetes and Alzheimer's disease.^[^ ^27^, ^28^, ^29^ ^]^ To test its inhibitory effects on MPC in macrophages, we employed stable‐isotope tracing metabolomics (**Figure** **3**a). We found that treatment with MSDC‐0160 or UK5099 did not reduce ^13^C‐glucose labeling of glucose‐6‐phosphate (G‐6‐P), dihydroxyacetone phosphate (DHAP) and lactate in macrophages (Figure 3b‐3d), indicating that aerobic glycolysis was not impaired. As expected, both compounds significantly reduced ^13^C‐glucose labeled TCA cycle metabolites, including citrate, α‐ketoglutarate (αKG), succinate and malate (Figure 3e‐3i), confirming that MSDC‐0160 is a potent inhibitor of MPC like UK5099. However, in contrast to UK5099, treatment of LPS‐primed BMDMs with MSDC‐0160 had no impact on IL‐1β release, as well as on the cleavage of pro‐caspase‐1 and gasdermin D upon nigericin stimulation (Figure 3j‐3l). Thus, UK5099‐mediated inhibition of the NLRP3 inflammasome cannot be attributed to effects on MPC.

Figure 3UK5099 inhibits the activation of NLRP3 inflammasome independent of MPC. a) A schematic diagram illustrating the carbon flux from labeled glucose in the cytoplasm to its catabolism by the TCA cycle in mitochondria. b to i) U‐[^13^C]‐glucose‐labelled glycolytic metabolites (b to d) and TCA cycle metabolites (e to i), as indicated, in LPS‐primed BMDMs treated with 50 µM MSDC‐0160 or 50 µM UK5099 for 2 hours (*n* = 4, mean ± SEM). j,k) Production of IL‐1β (j) and TNF‐α (k) from BMDMs stimulated with LPS and nigericin and treated with MSDC‐0160 or UK5099. Data are representative of three independent experiments (*n* = 4, mean ± SEM). l) Western blots of cell lysates (Lys.) and supernatants (Sup.) from BMDMs stimulated with LPS and nigericin and treated with MSDC‐0160 or UK5099. Data are representative of three independent experiments. m, n) Expression of *Mpc1* (m) and *Mpc2* (n) mRNA in *Mpc1^fl/fl^* and *Mpc1^ΔLysM^* BMDMs. Data are representative of at least ten independent experiments (*n* = 3, mean ± SEM). o) Western blot of Mpc1 and Mpc2 protein in *Mpc1^fl/fl^* and *Mpc1^ΔLysM^* BMDMs. Data are representative of at least ten independent experiments. p) A schematic diagram illustrating the design of the NLRP3 inflammasome assay with UK5099 in *Mpc1^fl/fl^* and *Mpc1^ΔLysM^* BMDMs. q) Production of IL‐1β from *Mpc1^fl/fl^* and *Mpc1^ΔLysM^* BMDMs stimulated with LPS and nigericin and treated with different concentrations of UK5099. Data are representative of three independent experiments (*n* = 4, mean ± SEM). r) Western blots of cell lysates (Lys.) and supernatants (Sup.) from *Mpc1^fl/fl^* and *Mpc1^ΔLysM^* BMDMs stimulated with LPS and nigericin and treated with different concentrations of UK5099. Data are representative of three independent experiments. One way ANOVA followed by Tukey's test was used to compare multiple groups (b to k). Unpaired Student's *t*‐test was used to test the differences between two groups (m, n). Two‐way ANOVA followed by Tukey's test was used to compare multiple groups (q). Differences were considered statistically significant at ^*^ *P* < 0.05, ^**^ *P* ≤ 0.01, ^***^ *P* < 0.001 and ^****^ *P* < 0.0001; NS, no significant difference.

**Figure:**
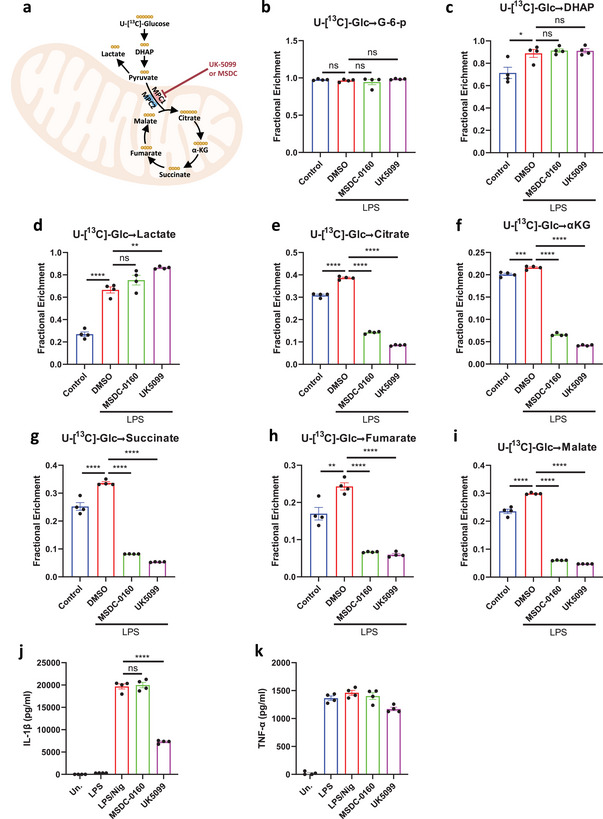

**Figure:**
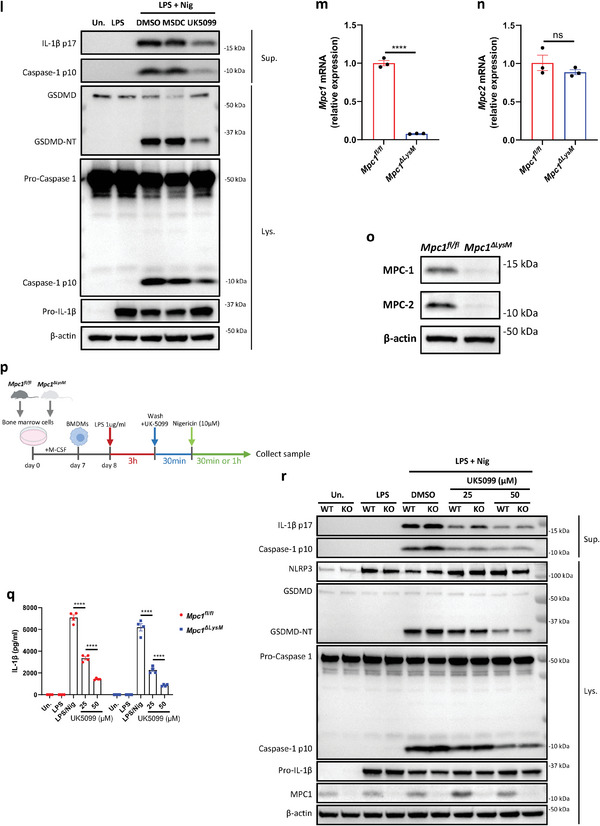

To further confirm this independence, we tested if UK5099 retains its inhibitory effect on NLRP3 in macrophages without MPC expression. To this end, we isolated BMDMs from *Mpc1^fl/fl^* and *Mpc1^ΔLysM^* mice as done previously.^[^ ^23^ ^]^ While MPC1 rather than MPC2 mRNA was deleted in *Mpc1^ΔLysM^* BMDMs (Figure 3m and 3n), neither MPC1 nor MPC2 protein were expressed as both subunits are known to be indispensable to form a stable MPC complex (Figure 3o).^[^ ^14^, ^15^ ^]^ LPS‐ primed *Mpc1^fl/fl^* and *Mpc1^ΔLysM^* BMDMs were then treated with UK5099 prior to nigericin stimulation (Figure 3p). Importantly, UK5099 decreased IL‐1β release to a similar extent in *Mpc1^fl/fl^* and *Mpc1^ΔLysM^* BMDMs (Figure 3q). Moreover, UK5099 similarly inhibited the cleavage of pro‐caspase‐1, pro‐IL‐1β and gasdermin D in *Mpc1^fl/fl^* and *Mpc1^ΔlysM^* BMDMs (Figure 3r). Collectively, this data demonstrates that UK5099 inhibits NLRP3 inflammasome activation independently of MPC.

### 2.4 UK5099 has no Effect on the Activation of the NLRC4 and AIM2 Inflammasomes

**Figure 4:**
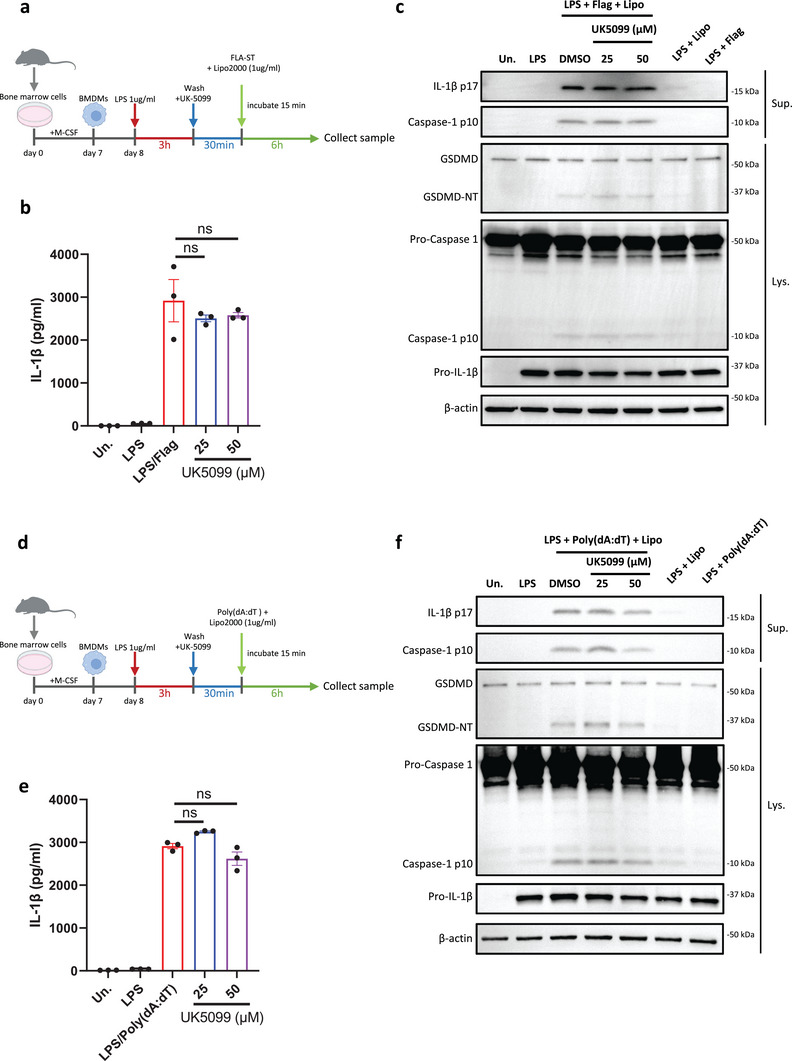
UK5099 has no effect on the activation of NLRC4 and AIM2 inflammasomes. a) A schematic diagram illustrating the design of the NLRC4 inflammasome assay with UK5099 in BMDMs. b) Production of IL‐1β from BMDMs stimulated with LPS and transfected flagellin and treated with different concentrations of UK5099. Data are representative of three independent experiments (*n* = 3, mean ± SEM). c) Western blots of cell lysates (Lys.) and supernatants (Sup.) from BMDMs stimulated with LPS and transfected flagellin and treated with different concentrations of UK5099. Data are representative of three independent experiments. d) A schematic diagram illustrating the design of the AIM2 inflammasome assay with UK5099 in BMDMs. e) Production of IL‐1β from BMDMs stimulated with LPS and transfected Poly(dA:dT) and treated with different concentrations of UK5099. Data are representative of three independent experiments (*n* = 3, mean ± SEM). f) Western blots of cell lysates (Lys.) and supernatants (Sup.) from BMDMs stimulated with LPS and transfected Poly(dA:dT) and treated with different concentrations of UK5099. Data are representative of three independent experiments. One way ANOVA followed by Tukey's test was used to compare multiple groups (b and e). NS, no significant difference.

We next evaluated whether UK5099 could inhibit the activation of other inflammasomes besides NLRP3. Firstly, LPS‐primed BMDMs were treated with UK5099, followed by the stimulation with purified flagellin, which activates the NLRC4 inflammasome (**Figure** **4**a).^[^ ^9^, ^10^ ^]^ We found that UK5099 did not reduce NLRC4‐stimulated secretion of IL‐1β and the cleavage of pro‐caspase‐1 and gasdermin D (Figure 4b,c). Next, we activated the AIM2 inflammasome by transfecting LPS‐primed BMDMs with the dsDNA analog poly(dA:dT) (Figure 4d).^[^ ^9^, ^10^ ^]^ Also in this setting, treatment with UK5099 between LPS priming and poly(dA:dT) transfection also did not reduce IL‐1β release, or the processing of pro‐caspase‐1 and gasdermin D (Figure 4e,f). Thus, UK5099 appears to selectively inhibit the NLRP3 inflammasome and not the NLRC4 and AIM2 inflammasomes.

### 2.5 UK5099 Abrogates the Interaction Between Mitochondria and NLRP3

Although the NLRP3, AIM2 and NLRC4 inflammasomes respond to different stimuli, all three sensor proteins share the same adaptor protein, ASC, and all impinge on the same downstream caspase‐1 and gasdermin D signaling pathway. As UK5099 selectively inhibits the activation of the NLRP3, but not the NLRC4 and AIM2, inflammasomes, we hypothesized that the molecular target of UK5099 is NLRP3 itself, either directly or indirectly, rather than its downstream signaling pathway.

Thus, we next investigated the underlying mechanism of how UK5099 might directly inhibit NLRP3. To this end, we first asked if UK5099 could directly bind to NLRP3. In our previous study,^[^ ^23^ ^]^ we screened UK5099‐bound proteins by using HuProt Proteome Microarray, which contains >21000 human proteins and covers ∼81% of the human proteome.^[^ ^30^ ^]^ We did not observe a direct bond between UK5099 and NLRP3, as indicated by similar signals between biotin and biotin‐UK5099.^[^ ^23^ ^]^ Furthermore, we utilized a cellular thermal shift assay (CETSA), which is a reliable approach to evaluate the direct biophysical interactions between small molecules and protein targets.^[^ ^31^, ^32^ ^]^ By doing so, we found that the degradation of NLRP3 increased with increasing temperature, but incubation with UK5099 did not further reduce NLRP3 levels as compared with a negative control, confirming that UK5099 is unlikely to direct bind to and activate NLRP3 (**Figure** **5**a). As UK5099 has a profound impact on the function of mitochondria,^[^ ^23^ ^]^ which plays an indispensable role in the activation of the NLRP3 inflammasome by directly recruiting NLRP3 or releasing essential macromolecules through the voltage‐dependent anion channel (VDAC) located in the mitochondrial outer membrane,^[^ ^33^, ^34^, ^35^ ^]^ we hypothesized that UK5099 interrupts the mitochondria‐NLRP3 interaction to suppress NLRP3 inflammasome activation. Indeed, inhibition of VDAC by VBIT‐4 reduced IL‐1β release but not TNF‐α production from BMDMs upon canonical NLRP3 inflammasome activation (Figure 5b,c). Of note, the inhibitory effect of UK5099 on NLRP3 was largely abolished in the presence of VBIT‐4, as indicated by similar levels of IL‐1β production, as well as the cleavage of pro‐caspase‐1 and gasdermin D, across the different doses (Figure 5d,e). However, VBIT‐4 treatment had no influence on the dose‐dependent inhibitory effects of MCC950 on IL‐1β release, as well as the processing of pro‐caspase‐1 and gasdermin D (Figure 5f to 5i), and MCC950 is known to directly bind the NACHT domain of NLRP3.^[^ ^2^, ^36^, ^37^ ^]^ Therefore, these data suggest that UK5099 indirectly inhibits NLRP3 via abrogating the mitochondria‐NLRP3 interaction.

Figure 5UK5099 interrupts the mitochondria‐NLRP3 interaction. a) Left: Western blots of the thermostability of NLRP3 following heat treatment at the indicated temperatures in the presence DMSO or 10 µM UK5099. Data are representative of three independent experiments. Right: Quantification of thermostable proteins whose signal intensity was normalized to the respective intensity at 37 °C (*n* = 3, mean ± SEM). b,c) Production of IL‐1β (b) and TNF‐α (c) from BMDMs stimulated with LPS and ATP and treated with UK5099 and Vbit‐4. Data are representative of three independent experiments (*n* = 4, mean ± SEM). d,e) Western blots of cell lysates (Lys.) and supernatants (Sup.) from BMDMs stimulated with LPS and ATP and treated with the combination of Vbit‐4 and UK5099. Data are representative of three independent experiments. f,g) Production of IL‐1β (f) and TNF‐α (g) from BMDMs stimulated with LPS and ATP and treated with MCC950 and Vbit‐4. Data are representative of three independent experiments (*n* = 4, mean ± SEM). h,i) Western blots of cell lysates (Lys.) and supernatants (Sup.) from BMDMs stimulated with LPS and ATP and treated with the combination of Vbit‐4 and MCC950. Data are representative of three independent experiments. Two‐way ANOVA followed by Tukey's test was used to compare multiple groups (b,c,f,g). Differences were considered statistically significant at ^***^ *P* < 0.001 and ^****^ *P* < 0.0001; NS, no significant difference.

**Figure:**
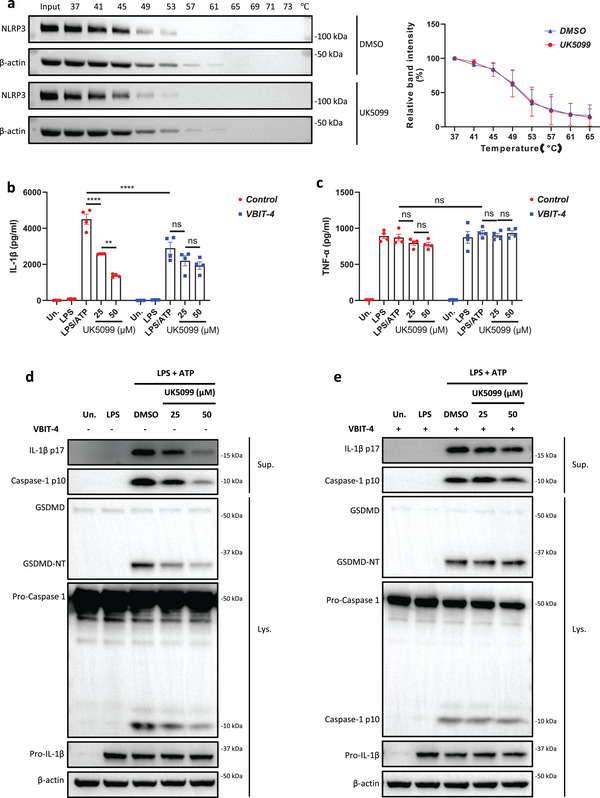

**Figure:**
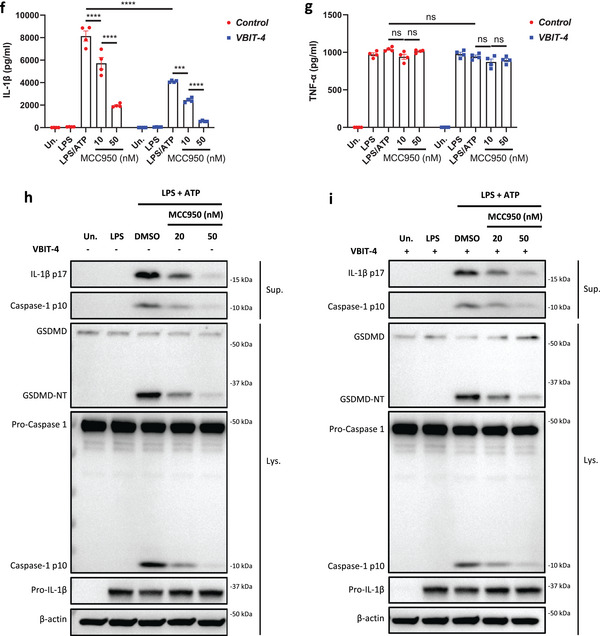

### 2.6 The Inhibitory Effect of UK5099 on the NLRP3 Inflammasome does not Require the Double Bond in UK5099

**Figure 6:**
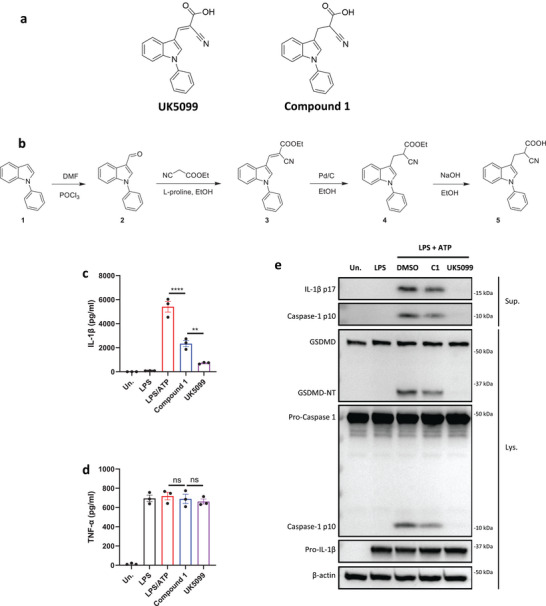
The inhibitory effect of UK5099 on NLRP3 inflammasome does not require the double bond in UK5099. a) The chemical structures of UK5099 and 2‐cyano‐3‐(1‐phenyl‐1H‐indol‐3‐yl) propanoic acid (Compound 1). b) The synthesis scheme of Compound 1. c,d) Production of IL‐1β (c) and TNF‐α (d) from BMDMs stimulated with LPS and ATP and treated with Compound 1 (C1) or UK5099. Data are representative of three independent experiments (*n* = 4, mean ± SEM). e) Western blots of cell lysates (Lys.) and supernatants (Sup.) from BMDMs stimulated with LPS and ATP and treated with Compound 1 (C1) or UK5099. Data are representative of three independent experiments. One way ANOVA followed by Tukey's test was used to compare multiple groups (c and d). Differences were considered statistically significant at ^**^ *P* ≤ 0.01 and ^****^ *P* < 0.0001; NS, no significant difference.

It has been hypothesized that UK5099 inhibits MPC through the reaction between its activated double bond and cysteine in MPC to form a covalent bond by using a Michael addition.^[^ ^12^, ^38^, ^39^ ^]^ Although this notion has been challenged by later studies, the existence of this double bond within the molecule is the major concern for the therapeutic applications of UK5099.^[^ ^11^, ^16^, ^40^ ^]^ We thus investigated if the inhibitory activity of UK5099 on the NLRP3 inflammasome relies on this double bond. To this end, we synthesized 2‐cyano‐3‐(1‐phenyl‐1H‐indol‐3‐yl) propanoic acid (compound 1) which has the same structure as UK5099 except that the double bond is replaced by a single bond (**Figure** **6**a,b) and tested its activity for inhibition of NLRP3 inflammasome activation. The loss of the double bond was confirmed by NMR (data not shown, but see the Methods section). Importantly, this newly synthesized compound, albeit weaker than UK5099, retained an ability to inhibit the NLRP3 inflammasome, as indicated by significantly reduced IL‐1β release, as well as decreased cleavage of pro‐caspase‐1 and gasdermin D (Figure 6c to 6e). Therefore, our data indicate that the inhibitory effect of UK5099 on NLRP3 inflammasome does not require the presence of the double bond in UK5099.

### 2.7 UK5099 Inhibits NLRP3‐Driven IL‐1β Release in Vivo

**Figure 7:**
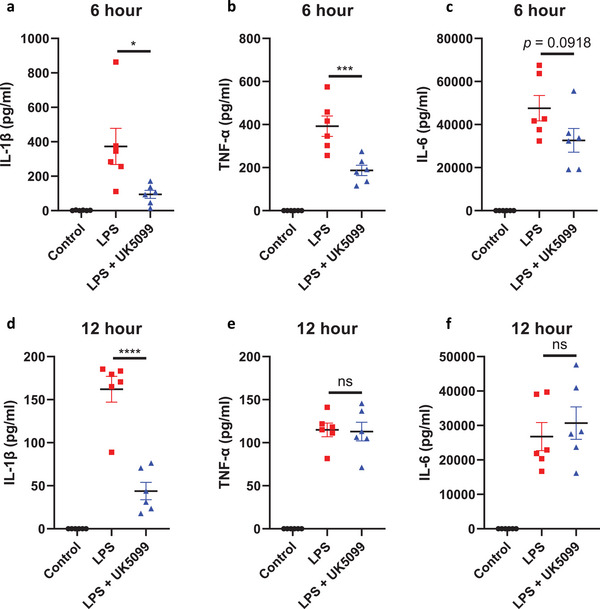
UK5099 reduces LPS‐induced inflammation in vivo. a to c) Serum levels of IL‐1β (a), TNF‐α (b) and IL‐6 (c) from C57BL/6 mice pretreated with 50 mg k^−1^g UK5099 or vehicle 6 h after i.p. LPS injection (15 mg k^−1^g) (n = 6 mice, mean ± SEM). d to f) Serum levels of IL‐1β (d), TNF‐α (e) and IL‐6 (f) from C57BL/6 mice pretreated with 50 mg k^−1^g UK5099 or vehicle 12 h after i.p. LPS injection (15 mg k^−1^g) (n = 6 mice, mean ± SEM). One way ANOVA followed by Tukey's test was used to compare multiple groups (a to f). Differences were considered statistically significant at ^*^ *P* < 0.05, ^***^ *P* < 0.001 and ^****^ *P* < 0.0001; NS, no significant difference.

Having shown that UK5099 is, among the various inflammasomes, a selectively inhibitor of the NLRP3 inflammasome in vitro, we next investigated the activity of UK5099 in vivo. To this end, we treated mice with 50 mg k^−1^g UK5099 before peritoneal injection of LPS to induce endotoxemia, which induces NLRP3‐dependent IL‐1β release in vivo.^[^ ^9^, ^10^ ^]^ Mice were sacrificed at 6 and 12 hours after LPS injection and proinflammatory cytokines production in the serum was determined. We found that a single dose of UK5099 significantly reduced IL‐1β production compared to LPS‐treated group at both 6 and 12 hours (**Figure** **7**a‐7f). The levels of TNF‐α and IL‐6 were lower in LPS+UK5099‐treated mice than those in the LPS‐treated group at 6 hours but recovered at 12 hours (Figure 7a‐7f), suggesting this early decrease in TNF‐α was likely secondary to NLRP3 inhibition.^[^ ^9^, ^24^ ^]^ This data demonstrates that UK5099 is sufficient to block NLRP3‐driven IL‐1β production in vivo. Of note, the treatment of UK5099 did not significantly improve the survival of mice after LPS challenge (Figure S1A, Supporting Information), which is consistent with the knowledge that the noncanonical rather than canonical inflammasome pathway dominates LPS‐induced sepsis.^[^ ^41^, ^42^ ^]^

We also compared the inhibitory ability of UK5099 with that of RRx‐001 which is a well‐established NLRP3 inhibitor. Unexpectedly, we found that mice treated with 50 mg k^−1^g RRx‐001 (i.p.) all died within 6 hours after LPS challenge, whereas mice that received the same dose of UK5099 remain stable, indicating the potential toxicity of RRx‐001. Therefore, we reduced the dose of RRx‐001 to 10 mg k^−1^g in accordance with a previous study,^[^ ^43^ ^]^ and found that UK5099 and RRx‐001 equally decreased the serum levels of IL‐1β mice after a challenge with LPS (Figure S1B, Supporting Information).

## 3 Discussion

IL‐1β is a key cytokine central to the inflammatory response and is involved in the development of numerous acute and chronic inflammatory diseases.^[^ ^44^, ^45^ ^]^ Directly targeting IL‐1β with a monoclonal antibody was shown to be effective for the treatment of cryopyrin‐associated periodic syndrome and systemic juvenile idiopathic arthritis, as well as for reducing the rate of recurrent cardiovascular events in patients with a history of myocardial infarction.^[^ ^46^, ^47^, ^48^ ^]^ However, patients who received this therapeutic had a higher incidence of fatal infection than those who received placebo, indicating that a certain degree of IL‐1β production might be required for the immune system to eliminate invading pathogens.^[^ ^48^ ^]^ Targeting the upstream NLRP3 inflammasome by specific inhibitors could avoid these shortcomings as it selectively reduces NLRP3‐mediated IL‐1β production but has no impact on other inflammasome‐dependent responses, such as NLRC4 and AIM2.^[^ ^9^ ^]^ Indeed, NLRP3 stands as one of the best therapeutic targets to treat human diseases driven by inflammation.^[^ ^1^, ^2^ ^]^ However, despite intense interest, there is currently no clinically approved NLRP3 inhibitor.

Here, we uncovered an MPC‐independent role of UK5099 as a selectively inhibitor of the NLRP3 inflammasome, with no effect on the AIM2 and NLRC4 inflammasomes. UK5099 effectively suppresses NLRP3‐mediated IL‐1β production in both mouse and human primary macrophages in vitro, as well as in an LPS‐induced endotoxemia model in mice in vivo. Therefore, UK5099 could serve as a potential new treatment for NLRP3 inflammasome‐related inflammatory diseases. More importantly, given that UK5099 inhibits both NLRP3 and MPC offers unique advantages of this small molecular compound. While targeting NLRP3‐driven immune response has been recognized as an effective therapeutic strategy to treat numerous inflammatory diseases, inhibiting MPC is also emerging as a promising approach for metabolic disorders, including type 2 diabetes, Alzheimer's disease and non‐alcoholic steatohepatitis.^[^ ^28^, ^29^, ^49^ ^]^ Thus, UK5099 has the potential to treat these diseases with higher efficacy than approaches solely inhibiting one of these targets, which merits further exploration, though as highlighted below, derivatives of UK5099 that only target one of these molecules may be more desirable.

Our data demonstrates that UK5099 inhibits NLRP3 inflammasome activation independent of its activity on MPC. This finding is not only supported by divergent effects of the MPC inhibitor MSDC‐0160, but also confirmed by genetic approaches. Indeed, a recent study found that genetic depletion or pharmacological inhibition of pyruvate dehydrogenase kinase (PDHK), which promotes the entry of glycolysis‐generated pyruvate into the TCA cycle, reduces NLRP3 inflammasome activation in both mouse and human macrophages.^[^ ^50^ ^]^ Moreover, inactivation of MPC by genetic depletion or by use of MSDC‐0160 even increased monosodium urate crystals‐induced NLRP3 inflammasome activation.^[^ ^51^ ^]^ While we did not observe an increase in NLRP3 inflammasome activation by MPC depletion or MSDC‐0160 treatment, both studies indicate that the entry of pyruvate into mitochondria is not a requirement for the activation of NLRP3 inflammasome.

The finding that UK5099 separately inhibits NLRP3 and MPC is of manifold significance. Firstly, the results of studies that used UK5099 to assess the role of MPC‐mediated metabolism in the differentiation, activation and cytokine production of immune cells need to be interpreted with caution.^[^ ^17^, ^22^ ^]^ Given the pivotal role of the NLRP3 inflammasome in multiple types of immune cells, genetic approaches need to be considered to reevaluate whether the effects of UK5099 treatment are bona fide dependent on MPC or in fact driven by NLRP3. Secondly, the current findings lead to the hypothesis that the inhibitory effects of UK5099 on MPC and NLRP3 are dependent, respectively, on separate core chemical structures of this compound. This independence should allow for the introduction of structural modifications to UK5099 that allow for a derivative to be a specific inhibitor for MPC or NLRP3. This is noteworthy because while inhibition of MPC has been shown to be a promising therapeutic for the treatment of several common chronic diseases,^[^ ^28^, ^29^, ^47^ ^]^ as indicated above, it also has the potential to cause heart dysfunction and other complications.^[^ ^52^, ^53^, ^54^, ^55^, ^56^, ^57^ ^]^ Therefore, structural modifications to preserve the inhibitory effect on NLRP3 while preventing targeting of MPC, or vice versa, would be conducive to promoting the clinical use of relevant derivative of UK5099. Indeed, the findings that UK5099 inhibits NLRP3 inflammasome activation independent of its double covalent bond, which might be required for the binding between UK5099 and MPC (albeit controversial),^[^ ^11^, ^16^ ^]^ further support this hypothesis.

### 3.1 Limitations of Study

Our study has some limitations. Firstly, although we demonstrated that UK5099 effectively inhibits the NLRP3 inflammasome activation both in vitro and in vivo, its performance on treating NLRP3‐driven diseases was not evaluated in this study. This is partly because there is inadequate information regarding the pharmacokinetic and toxicokinetic behavior of this compound,^[^ ^58^ ^]^ which is required to determine the dosage and routes of administration to achieve an effective in vivo concentration. Secondly, the interacting protein(s) or other molecules (e.g., lipid or nucleic acids) with UK5099 that are responsible for the inhibition of the NLRP3 inflammasome were not identified despite our demonstration that the mitochondria‐NLRP3 interaction is likely to be interrupted by this compound. It is also unknown how UK5099 suppresses the production of proinflammatory cytokines, such as TNF‐α, IL‐6 and IL‐12, when added before LPS stimulation in macrophages.^[^ ^17^, ^18^, ^19^, ^23^ ^]^ Further studies are needed to explore the pharmacological characteristics and potential target proteins of UK5099 to facilitate its usefulness in the treatment of MPC or NLRP3‐related diseases.

## 4 Experimental Section

### Mouse Strains

*Mpc1^fl/fl^* mice were generated by CRISPR/Cas9, which targets exon 3–5 of the *Mpc1* gene of mice in C57BL/6J background as described previously.^[^ ^23^ ^]^ *Mpc1^fl/fl^* mice were then crossed with Lyz2‐Cre transgenic mice (The Jackson Laboratory) to generate mice with a myeloid‐specific deletion of *Mpc1* (*Mpc1^ΔLysM^*). Both male and female *Mpc1^ΔLysM^* and their littermate control mice (*Mpc1^fl/fl^*) were used in this study. ASC reporter mice expressing ASC fluorescent protein (ASC‐citrine) were purchased from The Jackson Laboratory. Wild‐type C57BL/6J mice were obtained from Model Organisms Center (Shanghai, China). Experimental cells were prepared from 6‐ to 8‐week‐old female mice. All mice were bred and maintained under pathogen‐free conditions with ad libitum access to food and water. Mouse protocols were approved by the Tongji University Institutional Animal Use and Care Committee (IACUC).

### Generation of Mouse Bone Marrow‐Derived Macrophages

Bone marrow‐derived macrophages (BMDMs) were prepared as previously described.^[^ ^59^, ^60^, ^61^ ^]^ Briefly, mice aged 6‐ to 8‐weeks were individually euthanized in a CO_2_ chamber, and both the femur and tibia were collected. Bone marrow cells were then flushed out from the bone, resuspended and grown in RPMI‐1640 media (10% heat‐inactivated FBS and 1% penicillin and streptomycin) containing 20 ng/ml M‐CSF. Half the volume of fresh growth medium was added on day 4. After 7 days in culture, macrophages were harvested and plated for further experimentation.

### Generation of Human Monocyte‐Derived Macrophages

Human monocyte‐derived macrophages (hMDMs) were generated according to previous studies.^[^ ^9^, ^62^ ^]^ Firstly, peripheral blood mononuclear cells (PBMCs) were isolated from the fresh peripheral blood of healthy donors from Shanghai East Hospital by density gradient centrifugation using Ficoll‐Paque PLUS according to the manufacturer's protocol.^[^ ^63^ ^]^ Monocytes were then enriched from PBMCs using anti‐CD14 microbeads and magnetic‐activated cell sorting (Miltenyi). The monocytes were incubated in RPMI‐1640 media (10% heat‐inactivated FBS and 1% penicillin and streptomycin) containing 50 ng/ml recombinant human M‐CSF at 37C and 5% CO2. The human CD14+ monocyte‐derived macrophages were generated after 6 days in culture and were used for further experiments. This study was approved by the Ethical Committee of Shanghai East Hospital.

### Inflammasome Activation Assays

BMDMs were seeded at 1×10^6^ /ml and hMDMs were seeded at 5 × 10^5^/ml. Cells were primed the following day with Ultrapure LPS, E. coli 0111:B4 (1 µg/ml, InvivoGen) for 3 h. The medium was removed, washed with phosphate‐buffered saline (PBS) and then replaced with serum‐free medium (SFM) containing DMSO, UK‐5099 (0‐100 µM) (Sigma‐Aldrich), MSDC‐0160 (50 µM) (Cayman) for 30 min. The concentrations of DMSO were identical in each group (≤ 0.1%). Cells were then stimulated with 5 mM adenosine 5′‐triphosphate disodium salt hydrate (ATP) (Sigma‐Aldrich) for 30 min or 10 µM nigericin (InvivoGen) for 1 h to activate the NLRP3 inflammasome. VBIT‐4 (10 µM) (Selleck) was added for 16 h before LPS or NLRP3 activators challenge.^[^ ^34^ ^]^

To activate the AIM2 inflammasome, poly(deoxyadenylic‐thymidylic) acid sodium salt (Poly dA:dT) (InvivoGen) was first incubated with Lipofectamine 2000 (Thermo Fisher Scientific) for 15 min at room temperature and added at final concentration of 1 µg/ml to LPS‐primed cells for 6 hours. To activate the NLRC4 inflammasome, purified flagellin from *S. typhimurium* (Invivogen) was first incubated with lipofectamine 2000 (Thermo Fisher Scientific) for 15 min at room temperature, then the LPS‐primed cells were transfected with the mixture with the final concentration of flagellin at 1 µg/ml for 6 h.

### Western Blotting

Cells were lysed in RIPA buffer supplemented with Complete Mini EDTA‐Free protease inhibitor cocktail and phosphatase inhibitor cocktail (Roche). Protein concentrations of cell lysate were determined by Pierce BCA protein assay kit (Thermo Fisher Scientific) and equal amounts of total protein from each sample were used. To detect cleaved IL‐1β and caspase‐1, proteins were precipitated from the supernatant by adding 5 µL Strataclean Resin (Agilent Technologies) to 500 µL cell supernatant. The mixture was vortexed for 1 min and then centrifuged at 250 x *g* for 2 min at 4 °C. After removal of supernatant, the pellet was resuspended in RIPA lysis buffer. Samples were then boiled in SDS sample buffer for 5 mins at 95 °C, separated by SDS‐PAGE and transferred to PVDF membranes (Bio‐Rad). The membranes were blocked in TBS plus 5% nonfat dry milk (Bio‐Rad) for 1 h and then incubated with primary antibodies overnight at 4 °C. After three washes, the membranes were incubated with HRP‐linked secondary antibodies for 1 h at room temperature in TBS‐T plus 5% nonfat dry milk. ECL western blotting chemiluminescent substrates (Thermo Fisher Scientific) were added for 5 min after washing three times. Bands of interest were developed by using an autoradiographic film.

Primary antibodies used were Mouse IL‐1β (AF‐401‐NA) (1:800) (R&D system); Human IL‐1β (AF‐201‐NA) (1:800) (R&D system); Caspase‐1 p10 (ab179515) (1:1000) (Abcam); Mouse Cleaved Gasdermin D (10 137) (1:1000) (Cell Signaling Technology); Human Gasdermin D (39 754) (1:1000) (Cell Signaling Technology); β‐actin (ab8226) (1:1000) (Abcam); NLRP3 (15 101) (1:1000) (Cell Signaling Technology); MPC1 (14 462) (1:1000) (Cell Signaling Technology); MPC2 (46 141) (1:1000) (Cell Signaling Technology);

### Enzyme‐Linked Immunosorbent Assay

The concentrations of cytokines in cell culture supernatants and mouse serum were measured by enzyme‐linked immunosorbent assay (ELISA) according to the manufacturer's instructions (R&D Systems). Absorbance was read at 450 nm and 540 nm respectively to determine cytokines level.

### Assays of Apoptotic Cell Death

PI assays were conducted as previously described.^[^ ^64^ ^]^ Briefly, LPS‐primed cells in 96‐well plate were washed with PBS and cultured in a basal salt solution (120 mM NaCl, 4 mM KCl, 1.5 mM CaCl2, 1 mM MgCl2, 25 mM Hepes, 5 mM glucose at pH 7.4) containing 1 µg/ml PI (Molecular Probes) and different concentrations of UK‐5099 for 30 mins. After adding ATP or nigericin, fluorescence was measured kinetically on a SpectraMax i3x Multimode microplate reader (Molecular Devices) at 533/617 nm (excitation/emission) at 37 °C for 2 hours. Maximum PI uptake was obtained using 1% Triton X‐100. Relative PI uptake was calculated as: (sample‐background)/(maximum‐background)*100%.

Lactate dehydrogenase (LDH) release from cells was measured by the CytoTox 96 Non‐Radioactive Cytotoxicity Assay (Promega) following the manufacturer's instructions.

### Confocal Microscopy

BMDMs isolated from ASC reporter mice (ASC‐citrine BMDMs) were plated on chamber slides at 1 × 10^6^/ml. Cells were primed the following day with LPS for 3 hours. After washing with PBS, cells were treated with different concentrations of UK5099 for 30 mins, followed by stimulation with nigericin for 1 h. The cells were then washed with ice‐cold PBS three times and fixed for 15 min in fixation solution consisting of 4% paraformaldehyde (PFA, Sigma‐Aldrich) in PBS. After three times washing with PBST, nuclei were stained with DAPI (4,6‐diamidino‐2‐phenylindole) (Sigma‐Aldrich). Images were captured using a laser‐scanning confocal microscopy.

### Isotope‐Tracing Experiments

Metabolic tracing analysis of U‐[^13^C]‐glucose in BMDMs was determined by LC/MS as previously described.^[^ ^23^, ^61^ ^]^ Briefly, 2×10^6^ WT BMDMs were stimulated with or without 1 µg/ml LPS for 3 hours (priming). Cells were then washed with PBS and incubated in labeling medium (glucose‐free RPMI‐1640 medium supplemented with 11.1 mM ^13^C_6_‐glucose) containing DMSO, 50 µg/ml UK5099 or 50 µg/ml MSDC for 2 hours. After removal of culture medium, cells were washed three times with saline and flash frozen by rapid immersion of dishes in liquid nitrogen. Frozen cells were then scraped from culture dishes kept on ice, frozen in liquid nitrogen. On the day of sample processing, frozen cells were placed on ice and 400 µL of 80% (vol/vol) methanol (cooled to −20 °C) was added into each sample. The mixed contents were sonicated then centrifuged at 18000 x *g* for 15 min at 4 °C. The supernatant was collected and an aliquot from each was made for account for the protein concentration, then all the samples were lyophilized in a SpeedVac. On the day of LC–MS/MS acquisition, samples were resuspended in 20 µL of LC–MS‐grade water, and 5 µL of each metabolite sample was injected and analyzed with an ultrahigh‐pressure liquid chromatography‐triple quadrupole mass spectrometer ACQUITY UPLC‐Xevo TQ‐S (Waters Corp., Milford, MA, USA). Raw data files generated by UPLC‐MS/MS were processed using MassLynx software (v 4.1, Waters Corp., Milford, MA, USA)for peak extraction, integration, identification, and quantification of each metabolite. R language (v4.1.1) was used for subsequent statistical analysis.

Metabolites were quantified based on total ion count peak area of specific mass ions. To determine ^13^C‐labeling, mass information for known fragments of labeled metabolites was retrieved. These fragments contained either the whole or partial carbon skeleton of the metabolite.  For each fragment, the retrieved data comprised mass intensities for the lightest isotopomer (without any heavy isotopes, M+0), and isotopomers with increasing unit mass (up to M+6) relative to M0. These mass distributions were normalized by dividing by the sum of M0 to M6, and corrected for the natural abundance of heavy isotopes, using matrix‐based probabilistic methods as described and implemented in Microsoft Excel. ^13^C‐labeling data are expressed as fractional abundance of each isotopologue of a measured metabolite pool, or relevant enrichment of each metabolite. Data were from three biological replicates.

### In Vivo LPS Challenge/ Endotoxin‐Induced Model of Sepsis

Male and female C57BL/6J mice aged between 10 and 12 weeks were used for the in vivo LPS challenge experiment. Briefly, sex‐ and age‐matched mice were randomly assigned to control, LPS, LPS plus UK‐5099, or LPS plus RRx‐001 groups. Mice were injected intraperitoneally with 50 mg k^−1^g UK‐5099, 50 mg k^−1^g and 10 mg k^−1^g RRx‐001 or vehicle control (DMSO/PBS) 1 h before i.p. injection of 15 mg k^−1^g LPS *Escherichia coli* O111:B4 (Sigma‐Aldrich) to induce an endotoxemia model. Serum was collected at 6 h or 12 h after LPS injection and cytokine levels of IL‐1β, TNF‐α and IL‐6 were determined by ELISA. Mouse survival was monitored at various time intervals post‐LPS injection.

### Cellular Thermal Shift Assay

CETSA was performed according to the previous studies.^[^ ^31^, ^32^ ^]^ Briefly, BMDMs were primed with LPS for 3 h. After 3 times washing with PBS, cells were collected and resuspended in NP‐40 solution [50 mM Tris‐HCL (PH 7.4), 150 mM NaCl, 1% NP‐40, 1 mM PMSF], followed by five freeze−thaw cycles of liquid nitrogen. The lysates were then centrifuged at 15 000 x *g* for 20 min at 4 °C. The supernatants were collected and incubated with 10 µM UK5099 or DMSO (negative control) for 30 min at room temperature. The aliquots of the mixtures were heated individually on a heat block at 37 to 71 °C for 6 min and then cooled on ice. After centrifugation at 15 000 x *g* for 30 min at 4 °C, the supernatants were collected for Western blot analysis.

### Synthesis of 2‐cyano‐3‐(1‐phenyl‐1H‐indol‐3‐yl) Propanoic Acid

2‐cyano‐3‐(1‐phenyl‐1H‐indol‐3‐yl)propanoic acid 5 was synthesized according to the following scheme:

1‐phenyl‐1H‐indole‐3‐carbaldehyde 2 was synthesized with commercially available 1‐phenyl‐1H‐indole by the Vilsmeier−Haack reaction and subsequently transformed to the corresponding conjugated cyano ester 3 via ethyl 2‐cyanoacetate in the presence of L‐proline. The C = C double bond of α,β‐unsaturated cyano ester was reduced by Pd/C‐catalyzed hydrogenation reaction followed by Ester hydrolysis reaction to obtain the title compound 5.

**Scheme**:


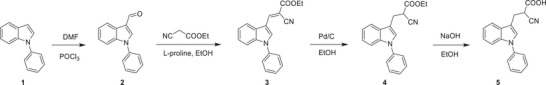


**Procedure**:

**Step 1**:


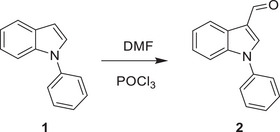


In a 100 mL round‐bottom flask equipped with a reflux condenser and a dropping funnel, 1‐phenyl‐1H‐indole (1 g, 5.2 mmol) was dissolved in DMF (2.3 mL, 31 mmol) with stirring. The mixture was cooled to 0 °C, and POCl_3_ (0.47 mL, 5.2 mmol) was added dropwise. On completion of the vigorous stage, the reaction mixture was heated for 2 h at 80 °C. The resulting red solution was poured into 10 mL of a warm saturated solution of Na_2_CO_3_ with stirring. The aldehyde was separated, washed with water, and dried in vacuo to give 1‐phenyl‐1*H*‐indole‐3‐carboxaldehyde 2 of 0.69 g as a colorless solid. Yield: 60%.

^1^H NMR (400 MHz, DMSO‐*d*_6_) δ 10.04 (s, 1H), 8.60 (s, 1H), 8.27‐8.19 (m, 1H), 7.75‐7.60 (m, 4H), 7.59‐7.50 (m, 2H), 7.35 (dd, *J* = 5.9, 3.0 Hz,2H). MS (+ESI): 222.1[M+H].


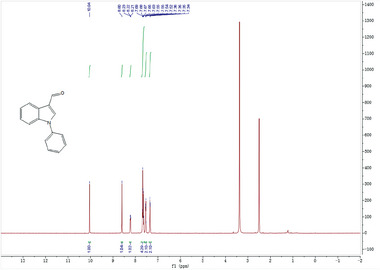


**Step 2**:


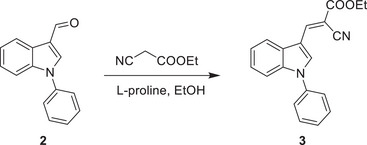


To a solution of 2 (0.69 g, 3.1 mmol) in EtOH (5 ml) was added ethyl 2‐cyanoacetate (0.44 ml, 4.1 mmol) and L‐proline (0.18 g, 1.2 mmol). The reaction was stirred at r.t. for 12 h and the yellow solid precipitated gradually. After completion of the reaction, ice‐cold water was added to the reaction vial. The solid was filtered and washed with water and dried to afford desired product 3 of 0.51 g as a yellow solid. Yield: 52%.

^1^H NMR (400 MHz, DMSO‐*d*_6_) δ 8.65 (d, *J* = 2.1 Hz, 2H), 8.13 (dd, *J* = 6.8, 2.7 Hz, 1H), 7.75‐7.63 (m, 4H), 7.63‐7.51 (m, 2H), 7.43‐7.28 (m, 2H), 4.32 (q, *J* = 7.1 Hz, 2H), 1.32 (t, *J* = 7.1 Hz, 3H). MS (+ESI): 317.0 [M+H]^+^.


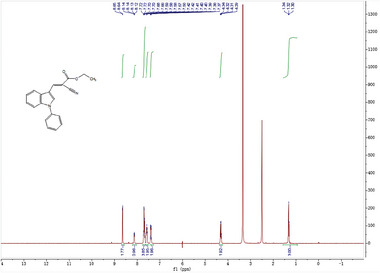


**Step 3**:


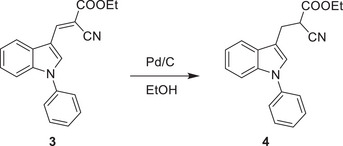


A solution of 3 (0.51 g, 1.6 mmol) in EtOH (5 ml) was added Pd/C (0.1 g), and the reaction was stirred at r.t. under an H_2_ atmosphere (balloon) for 12 h. Following the reaction mixture was filtered via celite pad and washed with EtOH and dried to afford desired product 4 of 0.45 g as a yellow oil. Yield: 88%.

**^1^H NMR**: (400 MHz, CDCl_3_): δ 7.66 (d, *J* = 4.0 Hz, 1H), 7.58 (d, *J* = 4.0 Hz, 1H), 7.56‐7.50 (m, 4H), 7.40‐7.38 (m, 2H), 7.26‐7.22 (m, 2H), 4.26 (q, *J* = 4.0 Hz, 2H), 3.88 (dd, *J* = 8.0 Hz, 4.0 Hz, 1H), 3.59‐3.56 (m, 1H), 3.49‐3.46 (m, 2H), 1.28 (t, *J* = 4.0 Hz, 3H). MS (+ESI): 319.0 [M+H]^+^.


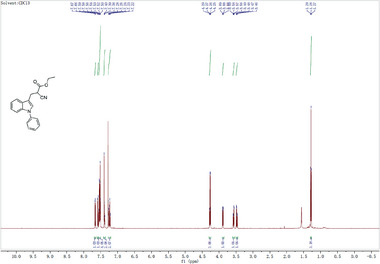


**Step 4**:


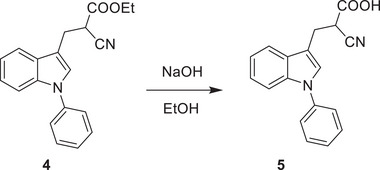


To a solution of 4 (0.4 g, 1.3 mmol) in THF (4 ml) and H_2_O (1 ml) was added NaOH (0.1 g, 2.6 mmol) and the reaction was stirred at r.t. for 3 h. Following the reaction mixture was acidified by adding con. HCl to pH 2∼3, then the mixture was filtered and washed with EtOH and dried to afford desired product 4 of 0.1 g as a light‐yellow solid. Yield: 28%.

**^1^H NMR**: (400 MHz, CDCl_3_): δ 7.65 (d, *J* = 8.0 Hz, 1H), 7.56 (d, *J* = 4.0 Hz, 1H), 7.54‐7.48 (m, 4H), 7.40‐7.36 (m, 2H), 7.27‐7.20 (m, 2H), 3.94 (dd, *J* = 8.0 Hz, 4.0 Hz, 1H), 3.60‐3.57 (m, 1H), 3.51‐3.47 (m, 2H). MS (+ESI): 291.0 [M+H]^+^.


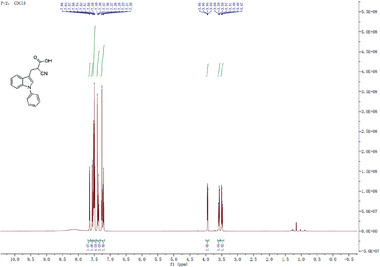


### Statistical Analysis

Results were presented as mean ± s.e.m. Unpaired Student's *t*‐test (two‐tailed) was used to test the differences between two groups affected by single variable, whereas one‐way or two‐way analysis of variance (ANOVA) with multiple comparisons test were used to compare multiple groups. Mouse survival rates were expressed as Kaplan‐Meier survival curves, and the difference between survival rates were analyzed using the log rank (Mantel‐Cox) test. Details of the *n* number can be found in the appropriate figure legend. All data were analyzed by GraphPad Prism software (version 9). *p < 0.05, **p < 0.01, ***p < 0.001, ****p < 0.0001. Detailed statistical value can be found in the figure legends.

## Conflict of Interest

The authors declare no conflict of interest.

## Author Contributions

L.R., M.C., J.Y., S.Z., and Z.L. contributed equally to this work. Conceptualization, F.W.; Methodology, F.W., L.R., Y.C., M.C., J.Y., C.Q., and S.Z.; Validation, F.W. and Y.C.; Investigation, L.R., M.C., J.Y., S.Z., Z.L., T.B., Q.Z., and M.S.; Writing – Original Draft, F.W.; Writing – Review & Editing, F.W., S.Z., and J.H.; Supervision, F.W., Q.L., and J.H.; Funding Acquisition, F.W., Q.L., and J.H.

## Supporting information

Supporting information

## Data Availability

The data that support the findings of this study are available from the corresponding author upon reasonable request.
