# Supplementary material for: UK5099 Inhibits the NLRP3 Inflammasome Independently of its Long‐Established Target Mitochondrial Pyruvate Carrier
Source: Adv Sci (Weinh). 2024 Jul 1;11(33):2307224. doi: 10.1002/advs.202307224 (PMC11434118; doi:10.1002/advs.202307224)
Supplement: Supplementary file 1 — Supporting information [file ADVS-11-2307224-s001.pptx]

## Slide 1
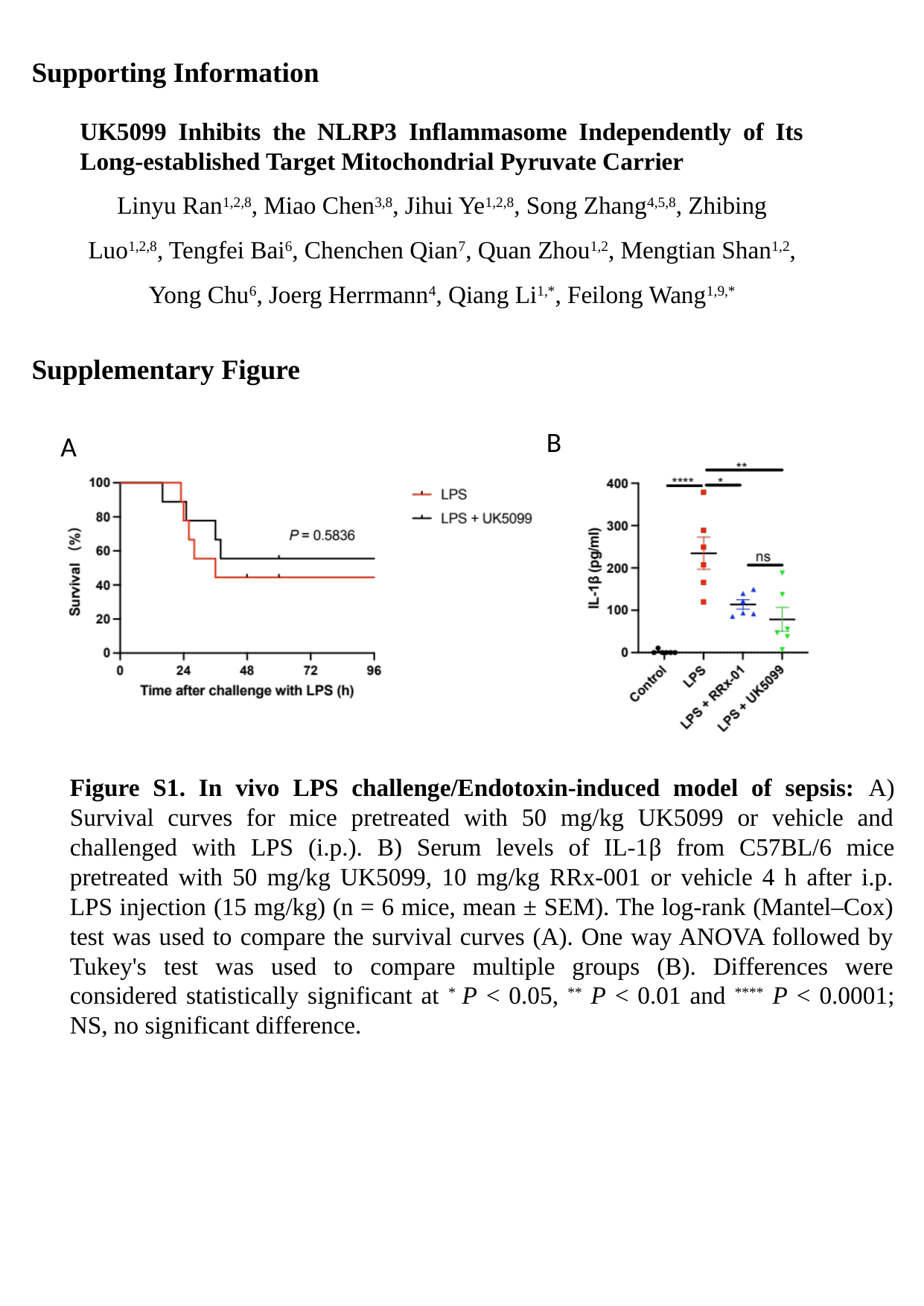

Supporting Information
UK5099 Inhibits the NLRP3 Inflammasome Independently of Its Long-established Target Mitochondrial Pyruvate Carrier
Linyu Ran1,2,8, Miao Chen3,8, Jihui Ye1,2,8, Song Zhang4,5,8, Zhibing Luo1,2,8, Tengfei Bai6, Chenchen Qian7, Quan Zhou1,2, Mengtian Shan1,2, Yong Chu6, Joerg Herrmann4, Qiang Li1,*, Feilong Wang1,9,*
Supplementary Figure
B
A
Figure S1. In vivo LPS challenge/Endotoxin-induced model of sepsis: A) Survival curves for mice pretreated with 50 mg/kg UK5099 or vehicle and challenged with LPS (i.p.). B) Serum levels of IL-1β from C57BL/6 mice pretreated with 50 mg/kg UK5099, 10 mg/kg RRx-001 or vehicle 4 h after i.p. LPS injection (15 mg/kg) (n = 6 mice, mean ± SEM). The log-rank (Mantel–Cox) test was used to compare the survival curves (A). One way ANOVA followed by Tukey's test was used to compare multiple groups (B). Differences were considered statistically significant at * P < 0.05, ** P < 0.01 and **** P < 0.0001; NS, no significant difference.
